# Iron’s Wake: The Performance of Quantum Mechanical-Derived Versus General-Purpose Force Fields Tested on a Luminescent Iron Complex

**DOI:** 10.3390/molecules25133084

**Published:** 2020-07-06

**Authors:** Valentin Diez-Cabanes, Giacomo Prampolini, Antonio Francés-Monerris, Antonio Monari, Mariachiara Pastore

**Affiliations:** 1 Université de Lorraine & CNRS, LPCT UMR 7019, F-54000 Nancy, France; antonio.frances@univ-lorraine.fr; 2 Istituto di Chimica dei Composti Organo Metallici (ICCOM-CNR), Area della Ricerca, via G. Moruzzi 1, I-56124 Pisa, Italy; 3 Departament de Química Física, Universitat de València, 46100 Burjassot, Spain

**Keywords:** iron complex, chemical environment, force field molecular dynamics, time-dependent density functional theory

## Abstract

Recently synthetized iron complexes have achieved long-lived excited states and stabilities which are comparable, or even superior, to their ruthenium analogues, thus representing an eco-friendly and cheaper alternative to those materials based on rare metals. Most of computational tools which could help unravel the origin of this large efficiency rely on ab-initio methods which are not able, however, to capture the nanosecond time scale underlying these photophysical processes and the influence of their realistic environment. Therefore, it exists an urgent need of developing new low-cost, but still accurate enough, computational methodologies capable to deal with the steady-state and transient spectroscopy of transition metal complexes in solution. Following this idea, here we focus on the comparison between general-purpose transferable force-fields (FFs), directly available from existing databases, and specific quantum mechanical derived FFs (QMD-FFs), obtained in this work through the Joyce procedure. We have chosen a recently reported Fe^III^ complex with nanosecond excited-state lifetime as a representative case. Our molecular dynamics (MD) simulations demonstrated that the QMD-FF nicely reproduces the structure and the dynamics of the complex and its chemical environment within the same precision as higher cost QM methods, whereas general-purpose FFs failed in this purpose. Although in this particular case the chemical environment plays a minor role on the photo physics of this system, these results highlight the potential of QMD-FFs to rationalize photophysical phenomena provided an accurate QM method to derive its parameters is chosen.

## 1. Introduction

Photoactive transition metal complexes (TMCs) characterized by long-lived (hundreds of picoseconds up to nanoseconds) metal to ligand charge transfer (MLCT) excited states have found successful application in different technological fields, spanning from natural and artificial photosynthesis [1,2,3,4], photovoltaic applications [5,6,7,8] to light-assisted medical therapies [9,10,11]. In the last decades, the exceptional development of time-resolved spectroscopies, allowing for femtosecond resolution of the different relaxation processes, covering UV/Vis, IR and also X-ray domains [12,13,14,15,16], have paved the way to a deeper investigation and a more detailed characterization of the photo-physics of TMCs. Undoubtedly, ruthenium polypyridyl complexes have dominated the scene, and they still retain unsurpassed record efficiencies in photovoltaic and photocatalytic devices [17,18,19,20,21,22,23]. Despite its successful and ubiquitous employment, ruthenium presents serious drawbacks, which potentially limit its large-scale applicability: toxicity and rarity. Finding Earth-abundant and eco-friendly alternatives to ruthenium complexes represents therefore a significant scientific and technological challenge. In the last years, increasing and intense research efforts have been addressed towards iron, which is copious, cheaper and environmental-friendly. Unfortunately, ultrafast relaxation to metal centered (MC) states followed by ground-state recovery is the dominant relaxation pathway in iron polypyridine complexes [24,25,26,27] hence precluding its use in operating photoactive devices. However, the synergic effect of deficiency of pyridine and -donation capacity of *N*-heterocyclic carbenes (NHCs) in pyridyl-NHC iron(II) complexes, has been proven effective in altering the energy order of the excited state manifold, inducing a destabilization of MC states with respect to MLCT ones and thus suppressing their accessibility. Thus, important breakthroughs in increasing the MLCT lifetime have been reported in the last years [28,29,30,31,32], spanning from tens of picoseconds [33,34,35,36] to the nanosecond time scale [37]. Even though simple concepts based on the ligand field strength [38] can provide general guidelines to increase the MLCT states lifetime of iron TMCs, a more subtle understanding of the various electronic, structural and environmental effects ruling the excited states relaxation process has been recognized as of pivotal importance [39,40]. Indeed, the fine understanding of the interplay between electronic and structural factors in iron complexes can significantly alter their photophysical outcome, that, from a molecular modeling point of view, requires a deeper exploration of the relevant potential energy surfaces (PES) and the spanned conformational space. As a most paradigmatic example, one can cite the reported effect of facial and meridional isomerism in influencing the lifetimes of MLCT states in bidentate pyridyl-NHC iron(II) complexes [35]. Comprehensive reviews concerning the application of computational tools to get insights into the excited states deactivation mechanisms in TMCs can be found in references [27,41,42].

To complement the need to systematically explore the excited states PES, here we want to focus our attention on the computational treatment of finite temperature and environment (solvent) effects on the electronic and optical properties of TMCs [43,44,45,46]. Indeed, when dealing with charge-transfer excitations, one can expect that the dynamic of the surrounding solvent molecules, responding to the change in the solute’s charge density, may influence both the energetics and the dynamics of the excited states [47,48,49,50,51]. To properly tackle this issue from a theoretical and computational point of view, one has to explicitly consider the solvent molecules into the simulation. Although, nowadays, full ab initio (DFT) molecular dynamic (MD) simulations have become feasible even for rather large systems [48,52,53,54] such methods are still unaffordable to study the structure and dynamics of solvated TMCs over sufficiently long time scales. Hybrid molecular mechanics (MM) and quantum mechanics (QM) schemes (QM/MM) can be useful to overcome limitations due to the size of the system, as the solute can be treated at QM level and the solvent molecules by means of classical force fields (FF) [55,56], however, still sub nanosecond timescale can only be simulated. Thus, classical techniques such as molecular dynamics (MD) [57,58], allowing for much longer simulations (>ns), remain the only viable approach to generate a meaningful statistical analysis of the solvent structure as well as of its response to the changes in the solute electronic distribution [59]. The reliability of classical MD simulations, however, in turn depends on the accuracy of the employed FF, that is on the potential energy functions used to approximate the system’s total energy as the nuclei move [57,60,61,62,63]. The standard approach in classical simulations is to consider the FF parameters as general and transferable between different systems belonging to similar classes of molecules. The introduction of different atom types and atom bonds, although complicating the parametrization procedure, represents a practical way to partially take into account different “chemical environments”. As a matter of fact, various widely employed state-of-the-art FFs exists for organic and biomolecular systems, implemented in commercial and open source packages (GROMACS [64], AMBER [65], TINKER [66],). If this strategy, and in particular the transferability, is mostly beneficial in the case of organic compounds, the situation is more complicated when TMCs are concerned, since practically no accurate transferable FF parameters, capable to describe the metal-ligand interaction and the coordination geometry, exist [67,68,69,70]. Hence, in contrast to the usual black box and universal approach, one should turn to specific and accurate parameterizations, purposely tailored on the system under investigation [69]. In this framework, a possibility particularly appealing in the case of the design of novel iron complexes, consists in resorting to QM-derived FFs (QMD-FF) [61,69,71,72,73,74,75,76,77,78,79,80,81,82,83], which can be parameterized specifically for the target system, solely based on ab initio purposely computed data, without the need of experimental measures, which might be of scarce availability, for further calibration. The loss of universality brought about by such approaches will be compensated by the possibility to finely tune the properties of the TMC, and hence provide a balanced description of its dynamic as well as of the interaction with the solvent, and to possibly account for the structural deformation induced by the different populated excited states. 

**Figure 1:**
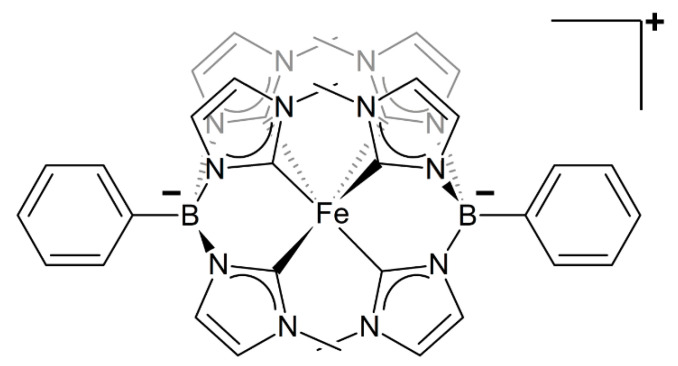
Chemical structure of the Fe^III^ complex [Fe(phtmeimb)_2_]^+^ studied in this work. The iron center is coordinated with six imidazole rings through six Fe-C bonds. The rings in the background are shown in light gray.

Here we evaluate and discuss the merits and shortcomings of these two striking opposite strategies by comparing the accuracy of a general-purpose, transferable, FF and a specifically tailored QMD-FF in predicting both the ground state structure and the room temperature Uv-Vis absorption of the octahedral Iron(III) complex with two phtmeimb ligands, were phtmeimb stands for [phenyl(tris(3-methylimidazol-1-ylidene))borate]^−^ (see Figure 1), recently reported by Wärnmark and co-workers [37,84], showing nanosecond ligand-to metal charge-transfer (LMCT) lifetime. 

For the definition of the QMD-FF, among others [69,71,72,73,74,75,77,78,79], we here resort to the Joyce protocol [78,85,86], already successfully employed to study the solvation features of octahedral TMCs [76,87], based on higher-level QM descriptors. Turning to the general-purpose FF, as an example of a transferable black-box approach, the standard General Amber Force Field (GAFF) method [88] was adopted whenever possible, by transferring all bonded FF parameters from the proper GAFF database, while retrieving the missing parameters (involving the metal and Boron sites) from supplementary literature databases, as described in Section 3.1.2. Initially, we have tested the reliability of both FFs to reproduce zero temperature structures using the QM ground state and X-ray diffraction (XRD) data geometries for validation. Afterwards, we performed MD simulations to get a realistic description of the chemical environment of the complex, in particular focusing on the interaction with the solvent (acetonitrile) and the counterion. In a final step, the absorption spectrum of the iron-III complex embedded in its environment was modeled by time dependent density functional theory (TD-DFT) calculations over a subset of the geometries obtained from the MD simulations, again carried out with both GAFF based FF (GbFF) and the QMD-FF, representing a statistically significant sampling of the spanned conformational space. Our findings support QMD-FF parametrization for the correct determination of the octahedral coordinated structure of the TMC and for a reliable sampling of the potential energy surface in proximity of the equilibrium structure. The latter effect is also reflected in the simulated optical spectra, for which a sampling performed using QMD-FF is mostly beneficial. However, TD-DFT calculations suggest that no major influence of the solvent shell structuration is observed, this in turn will point to an expected marginal role of the solvent in the differential stabilization of the MLCT and MC states for the specific case of the [Fe(phtmeimb)2]^+^ complex.

## 2. Results

In this section we present and compare the results obtained with a specifically tailored QMD-FF, as obtained by the Joyce procedure, (see computational details in Section 3.1.1) and a GAFF-based one (GbFF), built according to transferability considerations, whenever possible (see computational details in Section 3.1.2). Our analysis will be focused on two main aspects: (i) the structural properties of the Fe(III) complex and its interaction with the environment, and (ii) the optical properties obtained sampling the ground state conformational space with both approaches. In this regard the two subsections, respectively, deal with the static geometrical and optical properties, as obtained by means of MM energy minimization; and the effects of the solvent and the finite temperature as accounted by MD simulations.

### 2.1. Structural Properties from MM/MD Simulations

#### 2.1.1. Static Picture: DFT GS Geometry vs Molecular Mechanics

**Figure 2:**
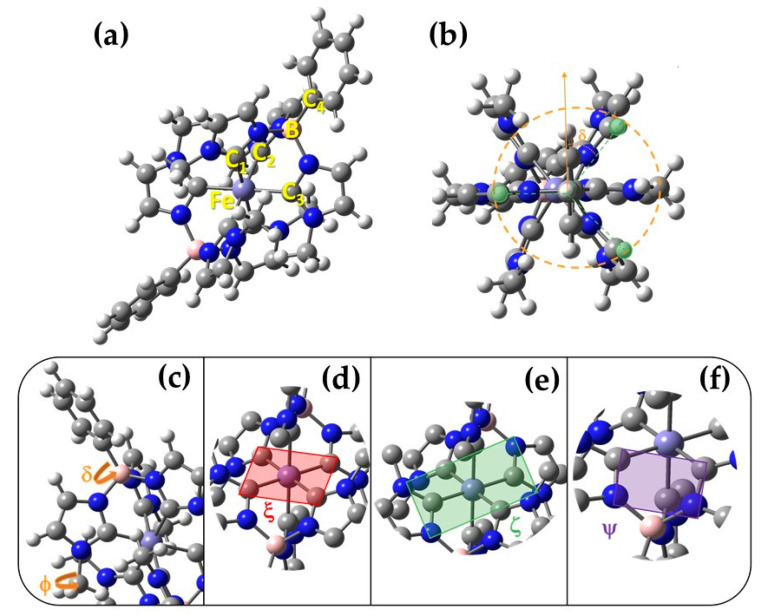
(**a**) DFT optimized GS structure of the iron complex in acetonitrile. Carbon, nitrogen, hydrogen, boron and iron atoms are displayed with gray, blue, white, pink and violet spheres, respectively and the atom labeling used in Table 1 is also reported; (**b**) Top view of the GS structure with a scheme showing the origin of the 60° periodicity of the δ torsional profile shown in Figure S1, with the imidazole hydrogens evidenced in green. Dihedral definition in the investigated iron complex: (**c**) flexible dihedrals δ and ϕ, ruling the rotation of the phenyl and methyl pendants, respectively; (**d**) stiff dihedral ξ, governing the coplanarity of four carbon atoms connected to the metal. ξ’ and ξ’’ can be defined exploiting the other two carbon quadruplets; (**e**) stiff dihedral ζ, governing the coplanarity of the four nitrogen atoms of two co-planar imidazole units. As for ξ, other two similar dihedrals can be defined exploiting symmetry; (**f**) stiff dihedral ψ, defining the plane formed by two nitrogen and two carbon atoms, as evidenced by the violet rectangle.

The complex studied in this work is formed by a Fe(III) central cation complexed with two monoanionic facial tris-carbene NHC ligands, forming a tridentate octahedral coordination sphere (see Figure 2a). Additionally, the two NHC ligands possess a tetrahedral geometry, where the tris-carbene ligands are connected to a phenyl ring via a formally anionic borate group. Since an accurate reproduction of the coordination geometry is expected to have a major importance, the geometrical parameters defining the nearly octahedral symmetry, or the metal-ligand bond lengths will play an essential role in tuning the photophysical response of TMCs.

To benchmark our zero temperature description and evaluate the accuracy of the calculated structural parameters, we have adopted as reference the bond and angles measured in single crystals of the Fe(III) complex grown in presence of hexafluorophosphate (PF_6_) and tetraphenylborate (BPh_4_) anions, which have been obtained via XRD measurements [37] (Table 1). In Table 1, XRD values are compared to the results obtained upon optimization of the doublet ground state (GS) at the QM (DFT) level and after MM energy minimizations, performed with both QMD-FF and GbFF, respectively. It is important to highlight that full XRD structures are not available in the reference work [37] (Table 1 already contains all reported XRD data), what makes impossible to get a direct comparison of theoretical and experimental geometrical parameters. Furthermore, solid-state geometries may also be affected by the inhomogeneous environment of the crystal leading to deviation from the ideal values that could be observed *in vacuo*. The XRD data confirm the near perfect octahedral coordination of the complex with ligand-metal-ligand angles equal to ~87° and metal-ligand distances close to 2 Å. The octahedral symmetry is only very slightly altered, most likely due to the steric repulsion between the hydrogen atoms belonging to the phenyl and imidazole rings, thus the bond parameters related with the three equivalent carbenes are not strictly equivalent. This effect, although weak, is more pronounced in the case of the XRD patterns obtained for the (PF_6_) single crystal, where one of the bond distances is shorter by 0.1 Å as compared to the other two equivalent bonds. This behavior is perfectly reproduced by the DFT GS optimization, which, in addition, provides geometrical parameters in remarkable agreement with the XRD data, as witnessed by the low values of the root mean square deviation (RMSD) for both distances (RMSD _[PF6]_ = 0.02 Å RMSD _[BPh4]_ = 0.04 Å) and angles (RMSD _[PF6]_ = 0.51° and RMSD _[BPh4]_ = 0.74°). Turning to the MM relaxed structures, QMD-FF optimized geometry shows identical values with respect to the DFT GS reference, as it could be expected due to the specificity of the DFT-derived parameterization of this FF, built for delivering optimized structures strictly equivalent to DFT. On the other hand, the bond parameters obtained by the GbFF procedure show larger deviation from both the XRD and the QMD-FF structures. Firstly, the absolute values of the metal-ligand angles are underestimated with respect to the XRD values, while the angles involving boron are overestimated, yielding to 5–6 times larger mean deviations (RMSD _[PF6]_ = 0.49 Å and RMSD _[BPh4]_ = 0.71 Å for the distances; RMSD _[PF6]_ = 3.21° and RMSD _[BPh4]_ = 3.01° for the angles). Moreover, and differently from the QMD-FF procedure, a standard FF parametrization is not considering any difference between the parameters of the three equivalent imidazole units. It therefore becomes apparent that GbFF is not able to capture the slight discrepancies existing in the three metal-ligand bonds and angles, as unambiguously observed in the XRD pattern.

**Figure 3:**
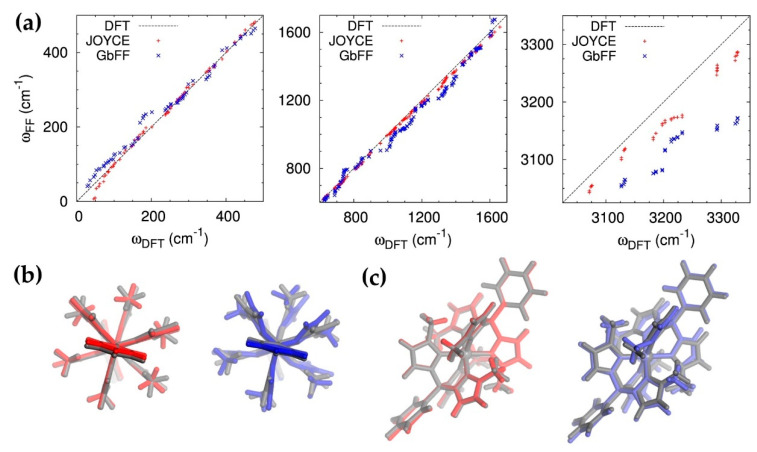
Comparison of the DFT vs Joyce/GbFF relaxed geometrical properties: (**a**) correlation plot between QM vs MM/Joyce and MM/GbFF structures vibrational frequencies as computed at the B3LYP*/6-311G* level of theory for low (0–500 cm^−1^), medium (600–1700 cm^−1^) and high (3050–3350 cm^−1^) frequencies from left to right top panels. It might be worth noticing that the x,y scale in last panel was increased with respect to the former, to better appreciate Joyce and GbFF differences; (**b**) Top and (**c**) side views of the overlap of QM (dark grey) and MM/Joyce (red) and MM/GbFF (blue) optimized geometries.

To evaluate the accuracy of the PES shape around the equilibrium region, we have also investigated the harmonic vibrational frequencies of the two MM relaxed complexes and compared them to the reference DFT values. The correlation between the QM (DFT) and MM (Joyce and GbFF) harmonic frequencies is presented in Figure 3a.

The agreement between the QM and MM normal mode frequencies is reasonably good for both types of FF implementations, however, the precision of Joyce’s calculated frequencies is globally higher with respect to GbFF, especially for high frequencies (ω > 3000 cm^−1^), where the discrepancies between the two force fields are more pronounced. In a second step, we have further quantified the accuracy of both FF methods in reproducing the DFT optimized geometry as witnessed by their RMSD.

To overcome spurious effects due to low-frequency, large-amplitude modes, such as –CH_3_ rotations, we decided to perform the analysis of the RMSD with respect to internal coordinates (bond distances, angles and dihedrals) compared to the reference DFT structure, which are collected in Table 2. As it is apparent from these values, the main deviations with respect to the reference geometries for both FF descriptors come from the dihedral angles, since the errors associated to bond and angles are extremely low, especially for the Joyce procedure, and can be considered negligible. In order to get a better visual representation of the differences obtained between MM and DFT optimized geometries, we have superimposed the former structures to the reference QM one, as represented in Figure 3b,c. In the case of Joyce, the main difference is located in the –CH_3_ dihedral (ϕ), which in some cases occupies a second local minima, thus contributing to the RMSD up to ∼120°. This is confirmed by looking at MM relaxed torsional energy scans of this dihedral (see Figure S1), obtained employing the QMD-FF as compared to their QM counterparts. The shape of the torsional curve registered for the ϕ dihedral is very similar to the well-known torsional profile of the CH_3_ rotation, with three minima at 180° and ±60°, with negligible barriers lower than 3 kJ/mol, clearly visible at 0° and ±120° (Figure S1). The δ relaxed scan presents conversely a more interesting behavior and differs from the one usually expected for a phenyl rotation, as for instance the inter-ring rotation in a biphenyl molecule [89]. Indeed, rather than presenting the usual flat minimum at ∼45°, with two similar barriers at 0° and 90°, and a periodicity of 90°, the torsional scan presents three minima (at ∼30°, ∼90° and ∼150°), thus showing a periodicity of 60°. This behavior can be rationalized by looking at Figure 2b, where a top view of the optimized conformer is shown, and the origin of the three-fold symmetry evidenced with orange and green lines. Indeed, the normal rotation of the phenyl pendant is prevented by the three hydrogen atoms (highlighted in green) of the neighboring imidazole units, whose C–H bond lies parallel to the B-phenyl axis that defines the δ rotation. In the bottom panel, the same analysis is presented for the stiff dihedrals. As a matter of fact, two degenerate QM minima appear in the ϕ profile at 60° and 180°, well reproduced by the MM curve. On the same foot, the three minima presented by the δ profile, at 30°, 90° and 150°, are also well reproduced, although with an overestimation of the barrier between the first two. This artifact can be traced back to the absence in the FF expression of explicit coupling terms between the δ torsion and the out-of-plane vibrations of the hydrogen atoms of the phenyl and the ligands, which at QM level may relax to lower the energy of the constrained conformation. Contrarily to Joyce, the overall structure obtained by GbFF minimization more significantly deviates from the QM reference. Indeed, while minima for the –CH_3_ dihedral (ϕ) profile are consistent with the QM structures, some groups such as the imidazole rings or the methyl chains appear folded. Several reasons can be attributed to the origin of this folded geometry such as the lack of a specific parametrization of the dihedral potentials of soft bond and their bending constants, hence once again pointing to the necessity of specifically tailored QMD procedure in case of complex structures presenting a rich density of degree of freedoms and a subtle coupling between their respective movements.

#### 2.1.2. Dynamic Effects and Interaction with the Solvent

In this section, we examine the capability of the different FF to efficiently sample the conformational space visited by the complex, also taking into account the effects of the solvent and counterions. This is achieved by performing classical MD simulations of the iron complex embedded with its corresponding PF_6_^−^ counterion in an acetonitrile solvent box. More details about the models and technical aspects of the MD simulations are given in Section 3.2. Firstly, we focused our analysis on the geometries of the different configurations reached by the complex along the MD run. In order to do so, we selected certain geometrical descriptors which will discriminate the different conformations explored by both the rigid structure of the octahedral scaffold and the flexible ligand groups. Indeed, despite the rather packed structure, a certain degree of flexibility should be expected, in particular for the rotation of the peripheral phenyl around the B-C bonds (dihedral δ), and the rotation of the methyl moieties tethered to one of the imidazole nitrogen atoms (dihedral ϕ). Both dihedrals, see panel c of Figure 2 for their visual description, should indeed be considered as flexible in the sense that large amplitude deviations from the equilibrium position (δ_0_ ~35°, ϕ_0_ ~40° as shown in Figure 2a should be expected, with consequent strong anharmonic effects. On the contrary, the coordination sphere around the metal is rather stiff, and large distortions from the octahedral arrangement should not be expected at room temperature. Moreover, the visual inspection of the computed normal modes, show other symmetric frameworks in the molecular structure contributing to the overall rigidity of the central molecular scaffold. To describe such modes and structure through simple internal coordinates, three triplets of stiff dihedrals have been introduced, namely ξ, ζ and ψ, as defined in the panels d–f of Figure 2, respectively. 

**Figure 4:**
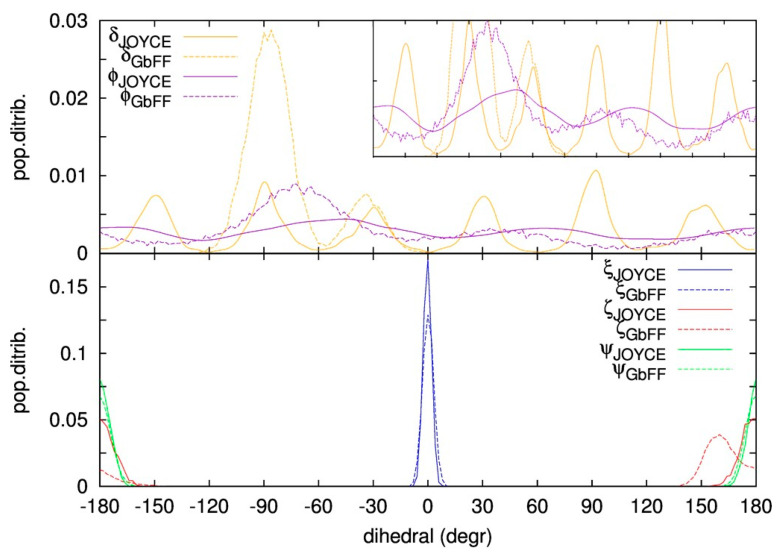
Distribution of flexible (**top**) and stiff (**bottom**) dihedral angles as defined in Figure 2c–f, during the MD runs performed with Joyce (continuous) and GbFF (dashed line) methods. In the top panel an insert caption of the graph with a lower y-scale is displayed on the top right part for the sake of visualization of the periodicity for the ϕ.

The distribution of both flexible (δ, ϕ, top panel) and rigid (ξ, ζ, ψ, bottom panel) dihedrals along the MD run are presented in Figure 4. Focusing first on the Joyce MD distributions, both the flexible and the rigid dihedrals did not show any artifact in their populations. In fact, as far as the two flexible angles are concerned, all minima are populated, while a negligible population is registered for the more repulsive QM regions. Consistently with the harmonic potential ruling their behavior, all distributions are more peaked around equilibrium values, indicating that the symmetric scaffold is in average preserved during the dynamics. Moreover, high temperature runs performed in vacuum confirmed this picture, and the population of the different minima almost reaches the same value as for the room temperature MD (see Figure S2). Observing now the distributions of the GbFF MD runs (dashed lines in Figure 4), the main differences with respect to their Joyce counterparts can be found in the flexible dihedrals. The ϕ dihedral distribution also follows a periodicity of 60°, but in this case their energy minima are slightly shifted (~10°) with respect to the Joyce runs, while keeping similar shape for both MD runs, hence indicative of similar energy barriers. Nonetheless, standard GbFF is not providing any specific parametrization for the ϕ dihedral and consequently, the energy barriers separating the different minima are unphysically high and consequently the system remains trapped close to the initial minima (δ_0_ ~ −90°) along the entire MD run. Regarding the rigid dihedrals, GbFF showed similar distributions compared with the Joyce MD runs, with the partial exception of the ζ minima which is shifted by 20° with respect to the ideal octahedral configuration. Notwithstanding that the octahedral central scaffold of the complex stays, on average, rigid also along the GbFF MD run, Joyce distributions are visibly stiffer than GbFF, hence pointing to a better preservation of the octahedral symmetry with respect to the general-purpose procedure. Finally, the similar distributions showed by the MD simulations performed in vacuum and in a solvent box (see Figure S3) confirm that no significant influence of the solvent on the structural and dynamic parameters should be outlined.

**Figure 5:**
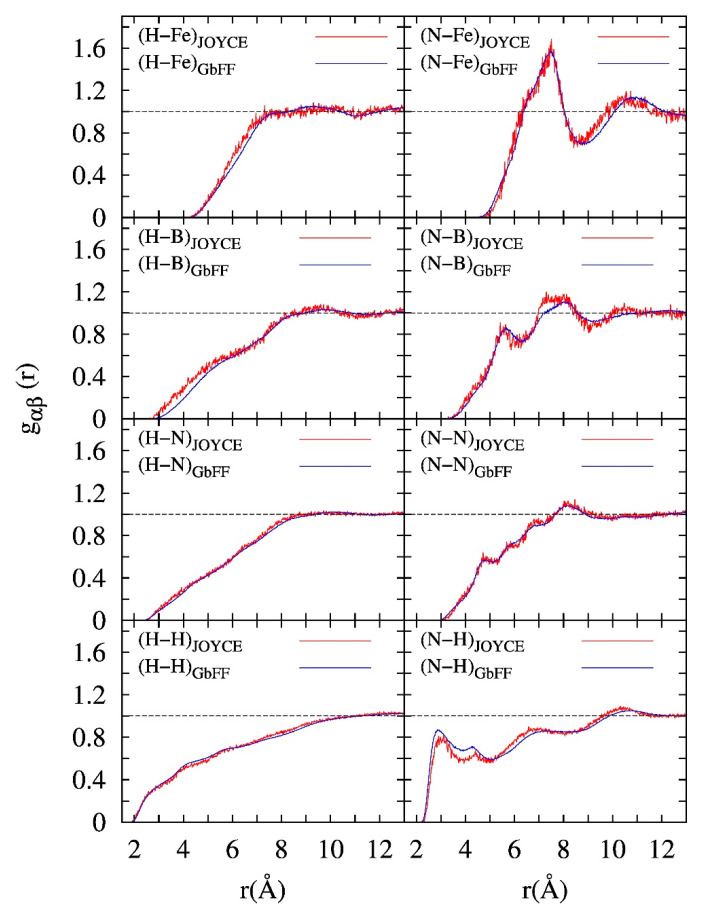
Atomic pair correlation functions g_αβ_, computed along the Joyce (red) and GbFF (blue) MD simulation runs, between the solute (β) atoms (Fe, B, N and H from –CH_3_ group) of the complex as indicated in the (α-β) legend, and either the N (left part) or H (right part graphs) atoms of the acetonitrile solvent (α).

The low influence of the solvent on the molecular structure is also confirmed by the analysis of the specific complex-solvent interaction as reproduced by both FFs. This has been achieved by computing representative atomic solute-solvent pair correlation functions, i.e. atom-atom correlation functions g_αβ_ (r_αβ_) defined between specific solute (α) and solvent (β) atoms. Selected results are displayed in Figure 5. The first clearly emerging result is that the interaction of the solvent with the metal complex is equally reproduced by both FFs. Moreover, this interaction is also independent of the choice of the atomic charges employed during the simulation (see Figures S4 and S5). By inspection of the two top panels of Figure 5, we can estimate that the first solvation shell is located at an average distance of about 7.5 Å from the metal ion, which in practice is far enough to not have a significant effect in the optical response of the complex. For instance, in the case of the much more solvent-dependent Ru(II)-polypyridine complex surrounded by dimethyl sulfoxide (DMSO) the first solvent shell is located at ca. 5.5 Å from the metal [76].

### 2.2. Optical Properties: Insights from TD-DFT Calculations

#### 2.2.1. Band Assessments from a Static Point of View

**Figure 6:**
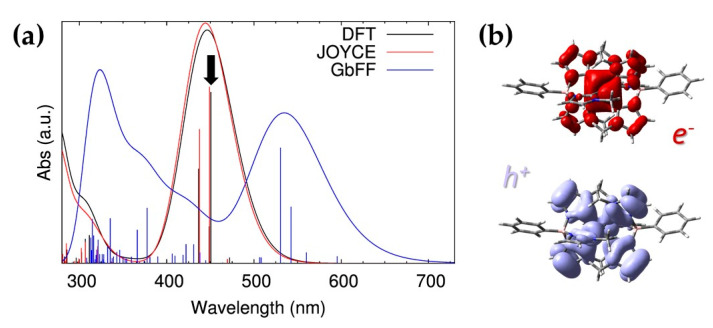
Absorption properties for the zero temperature structures: (**a**) Simulated spectra for the complex geometries obtained upon DFT (black), MM based Joyce (red) and GbFF (blue) energy relaxations. (**b**) NTOs of the main transition involved in the (^2^LMCT) band of the DFT optimized spectrum as highlighted by the black arrow on the left graph. Purple/magenta colors are used to visualize the holes/electrons isosurfaces, which were plotted with an isovalue equal to 0.02 a.u. All vertical excitations have been computed at the TD-B3LYP*/6-311G* level of theory.

Let’s start our analysis of the absorption properties of the complex by considering the first vertical transitions from the zero temperature geometries obtained by DFT and MM optimizations, in order to disentangle between static and dynamic effects. The simulated spectra for these three structures are presented in Figure 6a. 

In the visible region, the DFT and Joyce spectra are dominated by one single-band, centered at 450 nm, whose most intense vertical transitions are of (^2^LMCT) nature, whereas a narrower band appears in the UV region. As we discussed in the previous sections, the QM-derived parameterization of Joyce, and hence the almost total superposition of DFT and MM equilibrium geometries, are also reflected in the perfect agreement of the calculated vertical transitions in terms of both excitation energies and oscillator strengths. Differently, the spectrum obtained from the GbFF structure is sizably red-shifted, showing a (^2^LMCT) band centered at 535 nm with lower oscillator strengths as compared to the reference DFT.

The CT character of the (^2^LMCT) band has also been confirmed by computing the natural transition orbitals (NTOs) for the most intense low-energy transition, which clearly show that holes are mainly produced in the carbene ligands while electrons are localized in the central metal ion (see Figure 6b). The difference in the octahedral symmetry obtained with the three methods is also susceptible to affect the CT behavior of the (^2^LMCT) transitions. In order to quantify the CT character, we have calculated, for the three studied equilibrium structures, the Mulliken population analysis of the NTOs describing main (^2^LMCT) transition. By partitioning the obtained Mulliken charges over well spatially defined fragments, namely the central iron atom and each of the three NHC ligands, we can precisely define the spatial distribution, and the separation, of the holes and electrons (see Table 3). While in all cases an electron accumulation on the central iron atom is observed, a slight difference emerges in the case of the hole distribution. As it can be expected, the spatial distribution of holes and electrons for both Joyce and DFT structures remains very similar, being holes slightly asymmetrically distributed among the carbene ligands due to the above-mentioned breaking of the octahedral symmetry. Moving to the GbFF static structure, the NTO localization shows an asymmetric distribution of both holes and electrons, but in this case the CT is mainly localized in one of the ligands (CL_1_ in Table 3). The total amount of CT transferred to the metal cation is comparable in the three cases, being slightly smaller in the GbFF structure, which is again consistent with the deformation of the octahedral coordination.

#### 2.2.2. Dynamic Averaged Spectra in Solvent

**Figure 7:**
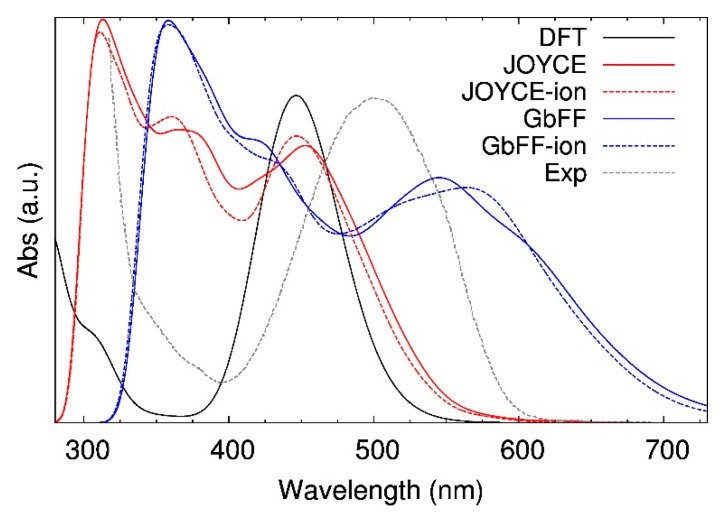
Calculated thermalized absorption spectra obtained for MD runs performed with Joyce (red) and GbFF (blue) methods, when employing solvent (continuous) and solvent+ion (dashed lines) QM/MM layers; together with the reference static DFT (black) and experimental (dashed grey line) spectra, which has been adapted from reference [37]. All vertical excitations have been computed at the TD-B3LYP*/6-311G* level of theory.

The effects of the thermal motion and the influence of the chemical environment (solvent and counterion) on the absorption properties of the iron complex have been accounted by analyzing the convoluted spectrum computed from the geometries extracted from both Joyce and GbFF MD simulations, according to the procedure detailed in Section 3.4. In Figure 7, both Joyce- and GbFF -based spectra arising from the MD runs are first compared to the static one, computed on top of the DFT zero-temperature geometry. Even though some absorption bands appear at higher energy regions, the averaged spectrum obtained with snapshots based on Joyce FF exhibits similar features with respect to the static DFT one, especially for the (^2^LMCT) band, which is also centered at 450 nm, whereas the band broadening associated to finite temperature effects is limited, the standard deviation for the (^2^LMCT) band center amounting to only 18.6 nm (see Table S1). This finding is in line with the rigidity of the chromophore core and with the large ion-solvent distances discussed in Figure 5, which point to a minor impact of the solvent on the absorption properties. Similar to the static spectra displayed in Figure 6, the averaged GbFF spectrum is substantially red-shifted with respect to the static DFT reference with the (^2^LMCT) band centered at 545 nm, while the broadening derived from the thermal motion now amounts to 32.1 nm. This result agrees with the stiffer distributions displayed by Joyce runs in the bottom panel of Figure 4. If we compare the averaged GbFF spectrum to its static equivalent in Figure 6, we only observe a 0.04 eV shift in the (^2^LMCT) band, thus also confirming that solvent and thermal effects are still rather marginal. The effects of the PF_6_ counterion have been investigated as well, by including this group in the QM/MM layer for those snapshots where the counterion remains at distance close enough to the metal ion, as explained in detail in Section 3.4. The thermalized spectra of the Joyce and GbFF MD run snapshots with counterion are represented with blue and red dashed lines respectively in Figure 7. Due to the small differences found when comparing these spectra with their solvent-only counterparts, we can safely conclude that the effects of the counterion in the absorption properties of the complex are also negligible.

Considering that accounting for both the solvent environment and finite temperature effects allows for reproducing the experimental conditions in which the absorption spectrum has been measured, the thermalized spectra obtained from Joyce and GbFF MD simulations are also compared in Figure 7 with the experimental spectra reported in reference [37], which is displayed with a dashed grey line in the graph. The experimental broad (^2^LMCT) band, centered at 502 nm, is not matched by any of the two investigated FFs: while Joyce overestimates this energy by 0.25 eV, GbFF underestimates it by 0.17 eV. Since we have shown that thermal and solvent effects have a negligible impact on the position of the (^2^LMCT) band, we can take the static DFT spectrum in implicit solvent (black solid line in Figure 7) as a reference to estimate the deviation from the experimental results. In this respect, the estimated blue-shift due to the TD-DFT approximations is of 0.27 eV, which obviously corresponds to the shift obtained for the Joyce spectrum, since the latter is almost coincident with the DFT one at 0 K (Figure 7) given the extremely similar geometries provided by the QMD-FF parametrization method (see Table 1). On the other hand, if we apply the same correction to the results obtained from the GbFF trajectory, the spectrum will now be much more red-shifted, significantly deviating from the experimental results. Hence, we can conclude that the relatively small red-shift apparent in Figure 7 for the GbFF results fortuitously comes from error cancellations. We can, thus, remark that the accuracy of QMD-FFs in estimating the absorption properties of iron complexes is mainly dependent of the choice of the method used to compute the excited states of the compound, which already points to the need of using high accurate and computationally feasible methods to model the optical properties of these materials. Note also that none of the spectra averaged from the MD trajectories reproduces the experimentally observed very low absorbance at 400 nm, i.e. in the region between the two main bands. This fact might be ascribed to a failure of TD-DFT in describing a band that is characterized by a complex mixture of both MLCT and LMCT bands. However, it is important to note that only Joyce spectra show an evident minimum at 400 nm, that is even deeper when the counterions are explicitly taken into account, which is in better agreement with the experimental results.

## 3. Materials and Methods

### 3.1. Force Field Parametrization

#### 3.1.1. Joyce Force Field

**Figure 8:**
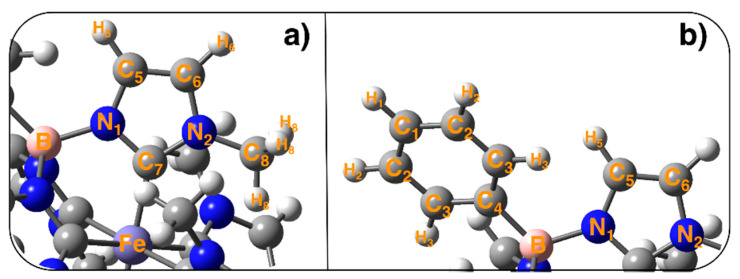
Atom types employed in all Joyce parameterization performed for the ground states of the investigated iron complex.

All QMD-FF parameters were obtained by means of a parameterization carried out with the Joyce code according to the standard protocol briefly outlined in the following. Further details can be found in the original papers and in most recent applications [76,78,85,86,87]. First, specific atomic types, based on symmetry and chemical equivalence considerations, were assigned to the target TMC. Concretely, apart from the iron (Fe) and boron (B) types, eight different atom-types were used for carbon, five for hydrogen and two for nitrogen: in every methyl-imidazole unit a separate atom-type (C_5_, C_6_, C_7_, C_8_, N_1_ and N_2_) was employed for each heavy atom and three different atom-types (H_5_, H_6_ and H_8_) for each hydrogen (see Figure 8 for visualization of the atom labelling). On the same foot, four different atom types (C_1_–C_4_) were employed for the carbon forming the phenyl rings, and three (H_1_–H_3_) for the hydrogens. Next, all possible stretching and bending coordinates were included in the MM description, together with several stiff dihedrals defined by quadruplets of heavy atoms were also included in the FF, as the ones ruling the planarity of the aromatic rings, the out-of-plane vibration of the aromatic hydrogens or the stiff dihedrals governing the octahedral structure of the metal-organic coordination (see panels d–f in Figure 2). Additionally, two kind of flexible dihedrals (δ and ϕ, see panel c of Figure 2), defined through one quadruplet of connected atoms and selected non-bonded distances between H_3_ and H_5_ atoms were also included. A model potential function is assigned to each considered internal coordinate, following the standard Joyce criteria, consisting in adopting harmonic potentials for all stretching, bending and stiff torsions, Fourier-like sums for the flexible dihedrals and Lennard-Jones functions for the non-bonded intra-molecular distances. The QMD-FF parameters were obtained by minimizing the standard Joyce objective function I:

(1) $$I=\overset{3N-6}{\underset{K\leq L}{\sum }}w_{KL}{\left(H_{KL}^{MM}-H_{KL}^{QM}\right)}^{2}+\overset{N_{geoms}}{\underset{k}{\sum }}w\prime_{kk}{\left(E_{k}^{MM}-E_{k}^{QM}\right)}^{2}$$

where the first sum runs over the target molecule’s QM normal modes, *H* is the Hessian matrix in the equilibrium geometry, *E_k_* the energy at the k-th geometry and *w_KL_* and *w*′*_k_* are appropriate weights. The usual Joyce two-step procedure [85,86] was adopted, with a first cycle which fits all harmonic parameters at once with respect to the computed QM Hessian and a second cycle, in which the harmonic parameters are fixed according to the Frozen Internal Rotation Approximation, [85] and the parameters for the flexible dihedral are parameterized against the QM torsional relaxed energy scans.

The parameterization was eventually carried out on a system of 403 redundant internal coordinates, by minimizing I as function of 398 independent parameters, obtaining a final standard deviation of 0.2 kJ/mol. The solvent (acetonitrile) and counterion are described through the OPLS parameters taken from the references [57,90,91] and [92], respectively. The topological (*.top) file of the Fe(III) complex with the QMD-FF parameters is provided in the Supporting Information. 

#### 3.1.2. GbFF Parametrization

The GbFF parameters for the metal complex, the solvent and the counterions have been built without any type of previous parameterization, only by employing tools and parameters which are already available in the literature. For the metal complex, all parameters involving the standard atoms (C, H, N) have been obtained with the antechamber program [93] available in the AMBER16 package suite of tools. The remaining parameters related with B and Fe atoms, which are not available in AMBER16 libraries [94], were calculated using different methods, aiming to keep the FF implementation as standard as possible. In the case of the B atom, all bonded parameters were obtained from DREIDING force field [95]. The equilibrium distance of the metal ion with the carbene ligands were calculated averaging the bond distances obtained by using the Z-matrix method as implemented in the Metal Center Parameter Builder (MCPB) Python program [96]. We considered an averaged value for the equilibrium Fe-C distances trying to reproduce the conditions of a standard FF parametrization, which makes no differences between the atom types of the six carbene ligands. The iron bond equilibrium force constants, bending angle and dihedral parameters were taken from a previous work dealing with octahedral Fe(II) complexes [97]. It is important to stress that the use of equilibrium Fe-C distances and C–Fe–C angles for the GbFF parametrization which are close to the values reported by XRD patterns is not implying a better description of the experimental absorption spectrum (see Figure S6). As far as non-bonded interactions are involved Van der Waals (VdW) parameters for boron were obtained from an early study dealing with borate groups [98]; while the parameters for the Fe atoms were adapted from reference [99]. Following the standard AMBER procedure, we estimated the atomic point-charges of our metal complex by computing the RESP charges calculated at the HF/6-31G* level of theory, as implemented in Gaussian 16 package [100]. For the description of the acetonitrile (ACN) solvent and the counterion (PF_6_^−^) we relied on the FF parametrization and atomic charges from references [101] and [102] respectively. Finally, all FF parameters were transformed into GROMACS file format by using the ACPYPE-AnteChamber PYthon Parser interface program [103].

### 3.2. MM/MD Simulations

The Molecular Dynamics (MD) simulations were conducted within GROMACS package suite of programs [64]. The MM optimized structure of the Fe complex was solvated into a cubic box containing ∼ 1000 acetonitrile molecules, previously equilibrated through NPT runs, at 1 atm and room temperature (298 K). A number of solvent molecules was also substituted with one PF_6_^−^ ion to assure electroneutrality. The final configuration resulted in a 50 × 50 × 50 Å box containing the Fe complex, its counterion and 979 acetonitrile solvent molecules. A first 100 ps simulation was performed in the NVT ensemble, using a time step of 0.1 fs with constrained applied to the Fe complex to freeze it to its equilibrium conformation. All velocities were assigned based on a random Maxwell-Boltzmann distribution at 298 K, and the temperature kept constant through the velocities rescale thermostat of Bussi et al. [104]. Thereafter, a longer 10 ns NVT equilibration was carried out from the final configuration of the previous run, removing the constraints on the solute.

This step was followed by a 2.5 ns equilibration in the NPT ensemble, at 298 K and 1 atm, coupling the thermostated system to a Parrinello-Rahaman barostat [105], with a time-step of 0.25 fs. Finally, to equilibrate the system in ambient conditions, a longer 5 ns run was further performed, in which all the C-H bonds were constrained through the LINCS algorithm, thus allowing for a time step of 1 fs. In Figures S7 and S8, a picture of the achieved equilibrated configuration is shown for a large and a specific region of the simulation box, respectively. Finally, a 10 ns production run was also performed in the NPT ensemble, at 1 atm and 298 K. The resulting trajectory was stored every 5 ps and used for the analysis discussed in the following.

### 3.3. DFT and TD-DFT Calculations

Quantum mechanical (QM) calculations were carried out at the density functional theory (DFT) level of theory, within the B3LYP* functional [106] with a non-standard exchange fraction (15%) and the 6-311G* basis set. This particular set of parameters was chosen due to the excellent description of the excitonic properties provided by this methodology with this type of TMCs [107,108,109]. Note that long-ranged correlated functionals (i.e. CAM-B3LYP [110]) which in theory should provide a better description of CT states, are not giving accurate results when treating the absorption properties of Fe complexes (see Figure S9). The static DFT geometry of the metal complex was obtained by optimization of its ground state in the gas phase. The optical properties were studied calculating the vertical excitation energies of the 35 lowest doublet excited states at TD-DFT level. Solvent effects were accounted implicitly by means of the C-PCM method [111]. All static QM calculations were performed within Gaussian16 package [100].

### 3.4. Thermally Averaged Absorption Spectra

Snapshots of the MD runs were stored every 2000 frames along the 10 ns production run, thus yielding a total of 201 geometries, which were used to calculate vertical transition energies at TD-DFT level using the same level of theory as previously discussed. We have first considered the configurations in which the effect of the counterion can be neglected, by extracting those snapshots where the distance between the metal ion and the counterion is larger than 14 Å. By applying this criterion, we obtained a total of ~115 structures. In order to study those cases where the counterion may induce a significant effect in the calculations, we have also analyzed those snapshots where the counterion is 10 Å apart from the metal center, obtaining further 80 structures. Hybrid QM/MM procedures are used to describe the effects of the molecular environment. Concretely, all solvent molecules surrounding the chromophore within a sphere of 14 Å and the counterion, if present, are included as point charges, whereas all solvent beyond 14 Å is also accounted for by a continuum polarizable medium, employing the C-PCM method. Finally, the thermalized averaged spectra were estimated by convoluting all vertical transitions with Gaussian function of half-width at full-length of 0.075 eV.

## 4. Conclusions

In this work, we have presented a theoretical study where we evaluated the accuracy of two different FF-based methods to reproduce the optical properties and environmental effects of a recently synthetized Fe(III) complex with luminescent properties. The direct comparison between the experimental XRD patterns, the reference QM GS and the structures obtained by MM energy minimizations with a QMD-FF and a general purpose FF procedure is a clear indication of the need of including QM effects in the derivation of the FF parameters for a realistic description of the octahedral coordination around iron complexes. Indeed, when comparing the reference QM GS with MM/Joyce derived structure, the latter FF method exhibits a precise description of the vibrational properties of the complex even at high frequencies, whereas the small differences found with respect to the reference GS geometry were ascribed to the population of different equivalent minima of –CH_3_ dihedral ϕ scan curves, which should not have any impact in the opto-electronic properties of the complex or the response of the structure to finite temperature effects. Completely different from the ones derived by Joyce, the geometries of the MM/GbFF optimized structure remained much less rigid when compared to the DFT geometry, with several dihedral angles far from the values of the GS reference. However, the structural parameters having the largest impact on the optical properties are those related to the octahedral coordination (the elongation of the ligand-metal bonds and the distortion of the octahedral geometry), hence eventual deficiencies of standard FF in their description will have the most critical effects.

MD simulations allow to precisely take into account the effects of finite temperature distortions from the equilibrium structure and of the chemical environment on the geometrical and optical properties of the complex. The geometry of the rigid central scaffold of the chromophore is not remarkably affected by the thermal motion. Hence, dynamical effects are not substantially modifying the absorption properties of the complex with respect to its static behavior. The distribution of the energy minima populated by Joyce and GbFF MD simulations differ mainly in the flexible dihedral δ related with the rotation of the phenyl group. While Joyce FF was able to reproduce the QM scan of this dihedral, the lack of a precise parametrization of the standard GAFF resulted in large energy barriers around a given minimum, thus providing unrealistic rigidity along the MD trajectory. In general, even if the rotation of the peripheral ligands will not affect substantially the absorption properties, it can be relevant in determining the luminescence quantum yield, since free rotations of the peripheral units may offer further non-radiative dissipative channels that could lower the luminescence efficiency.

Considering the effects of the chemical environment, both Joyce and GbFF MD showed that the first solvent shells remain considerably far (~7.5 Å) from the metal ion, also due to the rigidity of the central scaffold limiting its interaction with solvent molecules, resulting in a negligible effect of the chemical environment in the stabilization of the complex excited states. Lastly, the difference of the experimental absorption spectrum with the averaged Joyce/GbFF runs and the static QM spectra pointed to the deviations of the QM method employed to determine the excited state energies of the complex as the reason behind the discrepancies between the experimental and the thermally averaged spectra retrieved from Joyce MD runs.

To conclude, we emphasize the level of accuracy achieved by Joyce FF in describing the geometries of TMCs in realistic conditions with a computational cost which is orders of magnitude lower with respect to the QM method of reference. This methodology, combined to a suitable QM method for the calculation of the excited properties of TMCs, opens the door to perform further analysis which can be used to provide a deed understanding of the photo-physical phenomena measured experimentally (i.e., transient UV−Vis absorption spectra [59]), which are beyond the scope of this work.

## Figures and Tables

**Table 1:** Main geometrical parameters (bond distances and angles) defining the octahedral coordination of the complex, as obtained from the QM (DFT), MM (Joyce and GbFF) relaxed structures and from XRD measurements performed in [PF_6_] and [BPh_4_] counterion single-crystals, as reported in ref. [37]. The adopted atom labeling is depicted in Figure 2a. The level of theory adopted for the DFT calculations was B3LYP*/6-311G* (see Section 3.3).

	Atoms	DFT	Joyce	GbFF	XRD [PF_6_] ^1^	XRD [BPh_4_] ^1^
	Fe-C1	2.041	2.040	2.055	2.008	1.984
Bond distances (Å)	Fe-C2	2.027	2.026	2.052	2.002	1.979
	Fe-C3	1.998	1.999	2.048	1.979	1.971
	Fe-B	3.215	3.215	3.421	3.202	3.182
Bond angles (°)	C1-Fe-C2	85.57	85.64	82.31	86.49	86.47
	C1-Fe-C3	86.77	86.67	83.36	87.00	86.86
	C2-Fe-C3	87.47	87.53	84.52	87.24	86.86
	Fe-B-C4	173.4	173.4	175.5	173.7	174.4

^1^ Data taken from Kjær et al. [37].

**Table 2:** RMSD values of the internal coordinates for the MM relaxed geometries with Joyce and GbFF methods, which are obtained by taking the DFT GS geometry as reference.

FF Method	Bond Length (Å)	Bending Angle (°)	Dihedrals (°)
Joyce	0.00	0.07	15.89
GbFF	0.03	2.75	22.13

**Table 3:** Excitonic properties for the lowest-energy most intense transition involved in the (^2^LMCT) band of the static DFT, Joyce and GbFF simulated spectra represented in Figure 6: state involved (D*_i_*), wavelengths (nm) and energies (eV, within parenthesis), oscillator strengths (*f*); and fraction of the hole (*h*^+^) and electron (*e*^−^) localizations on the central metal ion (Fe) and equivalent carbene ligands (CL*_i_*) fragment as obtained from Mulliken population analysis of the NTOs. Note that the hole/electron fraction localized along the boron atoms and phenyl groups are not reported here due to their negligible values. All excitonic properties have been estimated at the TD-B3LYP*/6-311G* level.

Method	State	λ(nm)	*f*	*Fragment*	*h* ^+^	*e^−^*
				Fe	0.007	0.328
				CL_1_	0.306	0.188
DFT	D_4_	451 (2.75)	0.071	CL_2_	0.266	0.3
				CL_3_	0.414	0.177
				ΣCL	0.985	0.666
				Fe	0.008	0.332
				CL_1_	0.306	0.193
Joyce	D_4_	449 (2.76)	0.073	CL_2_	0.263	0.292
				CL_3_	0.414	0.176
				ΣCL	0.984	0.661
				Fe	0.018	0.31
				CL_1_	0.454	0.169
GbFF	D_7_	531 (2.34)	0.048	CL_2_	0.24	0.246
				CL_3_	0.271	0.253
				ΣCL	0.965	0.668

## Supplementary Materials

The following are available online. Figure S1: QM vs QMD-FF MM δ and ϕ dihedral scans, Figure S2: dihedral distributions for MD runs performed on the isolated Fe complex at 298 K and at 1000 K, Figure S3: MD run dihedral distributions in vacuum and solvent environments, Figure S4 and Figure S5: atomic pair correlation functions computed with different atomic charge descriptions along the Joyce and GbFF MD simulation runs respectively, Figure S6: simulated spectra for the complex geometries obtained upon the scan performed along the coordinates dictating the octahedral coordination, Figure S7 and Figure S8: large and specific regions for the equilibrated solvation box employed in the MD simulations, Figure S9: comparison of the simulated static spectra obtained with B3LYP* and CAM-B3LYP functionals, Table S1: statistical data taken from the (^2^LMCT) band centers distribution for the TD-DFT simulated spectra of the snapshots from the MD run, topological (*.top) file of the complex with the QMD-FF parametrization.
