# Supplementary material for: Iron’s Wake: The Performance of Quantum Mechanical-Derived Versus General-Purpose Force Fields Tested on a Luminescent Iron Complex
Source: Molecules. 2020 Jul 6;25(13):3084. doi: 10.3390/molecules25133084 (PMC7411876; doi:10.3390/molecules25133084)
Supplement: Supplementary file 1 [file molecules-25-03084-s001.pdf]

molecules

Supporting Information:

Iron's Wake: The Performance of Quantum Mechanical-Derived Versus General-Purpose Force Fields Tested on a Luminescent Iron Complex

Valentin Diez-Cabanes [1],* Giacomo Prampolini [2],* Antonio Francés-Monerris [1,3], Antonio Monari [1],* and Mariachiara Pastore [1],*

[1] Université de Lorraine & CNRS, LPCT UMR 7019, F-54000 Nancy, France; antonio.frances@univ-lorraine.fr (A.F-M.)

[2] Istituto di Chimica dei Composti Organo Metallici (ICCOM-CNR), Area della Ricerca, via G. Moruzzi 1, I-56124 Pisa, Italy;

[3] Departament de Química Física, Universitat de València, 46100, Burjassot, Spain

* Correspondence: valentin.diez@hotmail.com (V.D-C), antonio.monari@univ-lorraine.fr(A. M.), mariachiara.pastore@univ-lorraine.fr (M.P.) ; giacomo.prampolini@pi.iccom.cnr.it (G. P.)

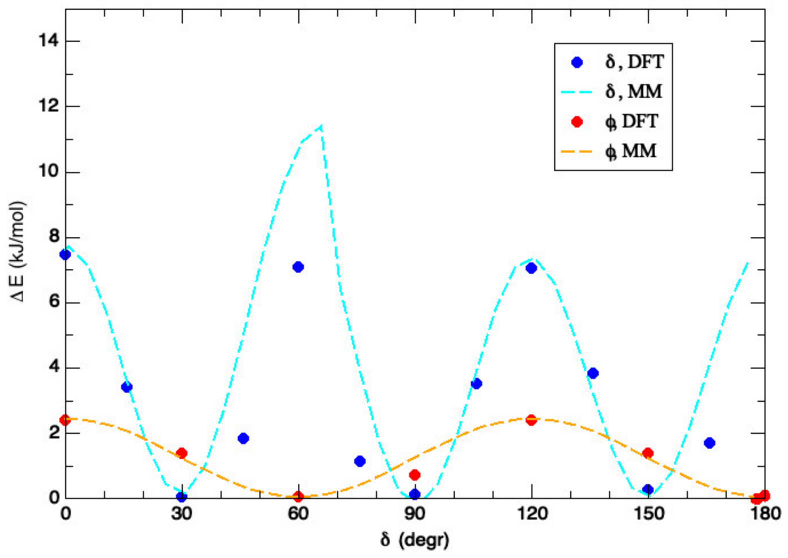

Figure S1. Comparison between QM (solid symbols) and MM (dashed lines) relaxed energy scans for the flexible δ (blue) and ϕ (red) dihedrals.

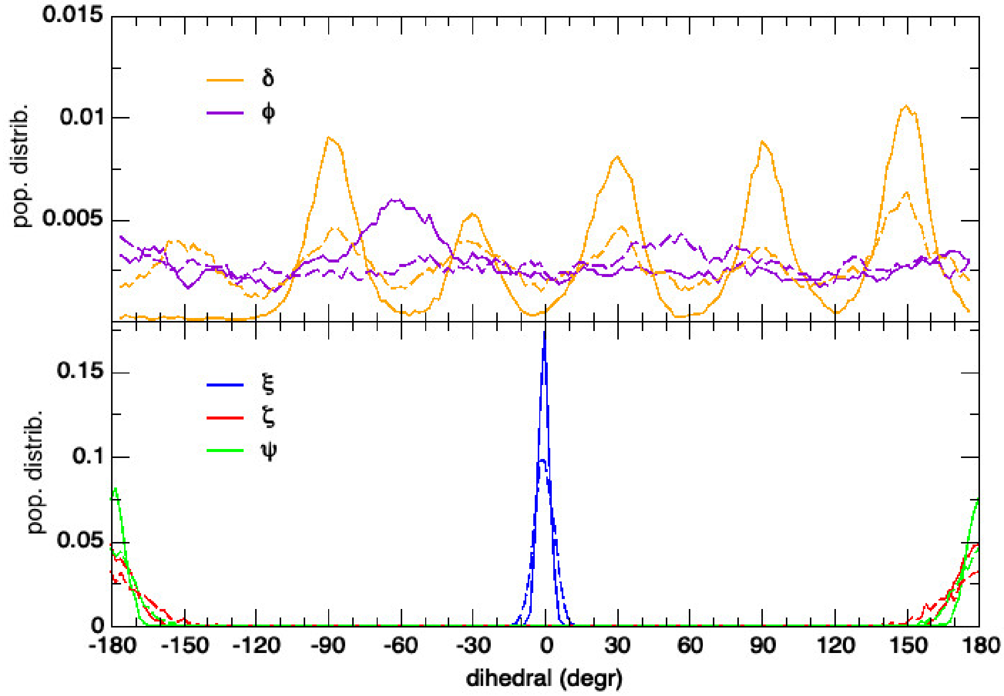

Figure S2. Distribution of flexible (top) and stiff (bottom) dihedral, defined in Figure 1, during an NVT-MD run performed on the isolated Fe complex at 298 K (solid lines) or at 1000 K (dashed lines).

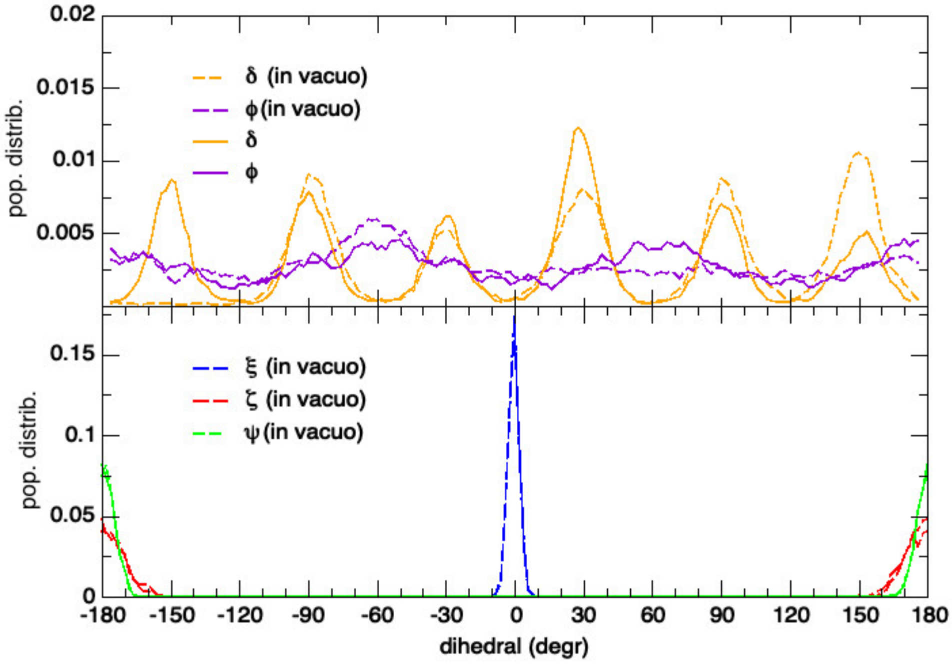

Figure S3. Distribution of flexible (top) and stiff (bottom) dihedral, defined in Figure 1, during the 10 ns NPT-MD production run performed on the solvated Fe complex at 298 K and 1 atm (solid lines) or in vacuum at the same temperature (dashed lines); by employing the QMD-FF parameters.

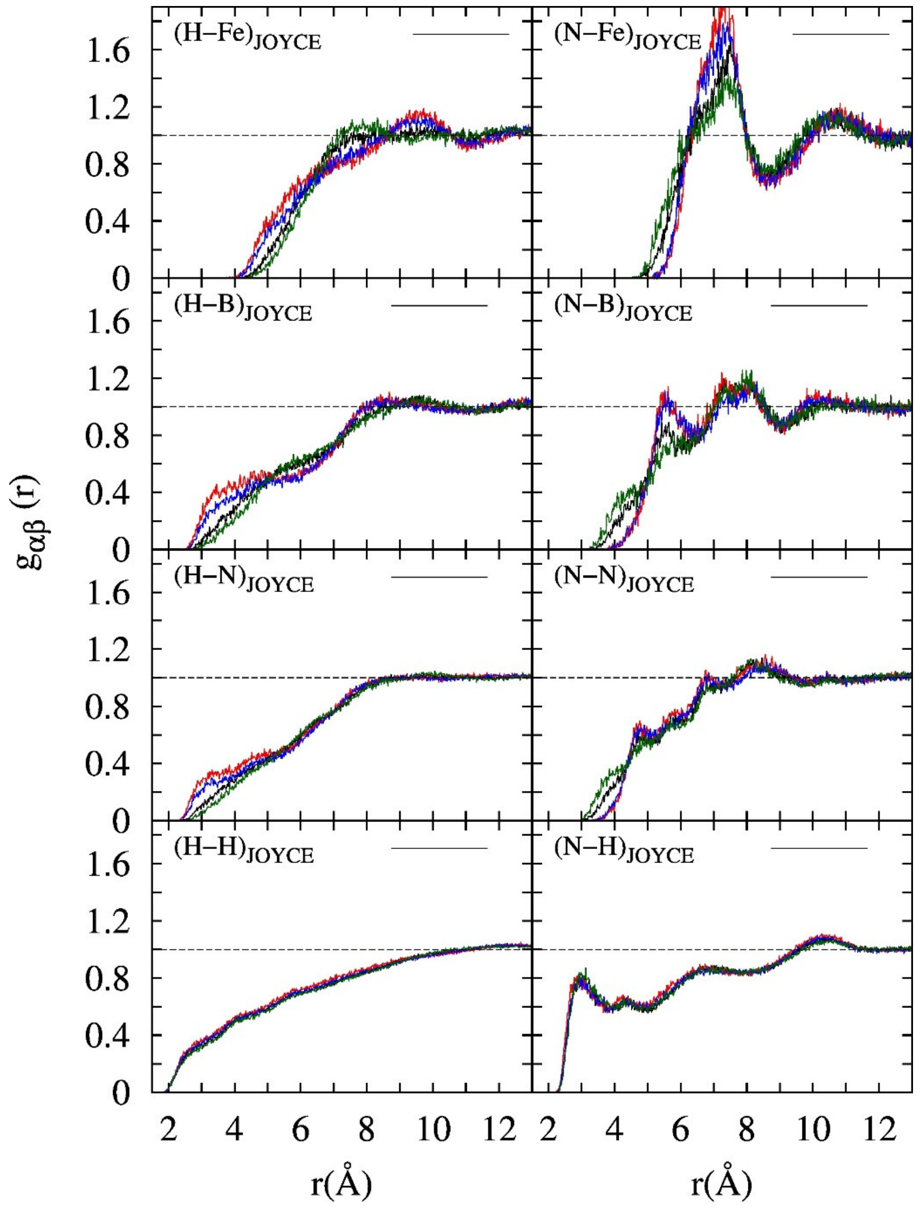

Figure S4. Atomic pair correlation functions $g_{\alpha\beta}$, computed with RESP (black), NPA (red), CM5 (blue) and ChelpG (green) atomic charges along the JOYCE MD simulation runs, between the solute (β) atoms (Fe, B, N and H from $-CH_3$ group) of the complex as indicated in the (α-β) legend, and either the N (left part) or H (right part graphs) atoms of the acetonitrile solvent (α).

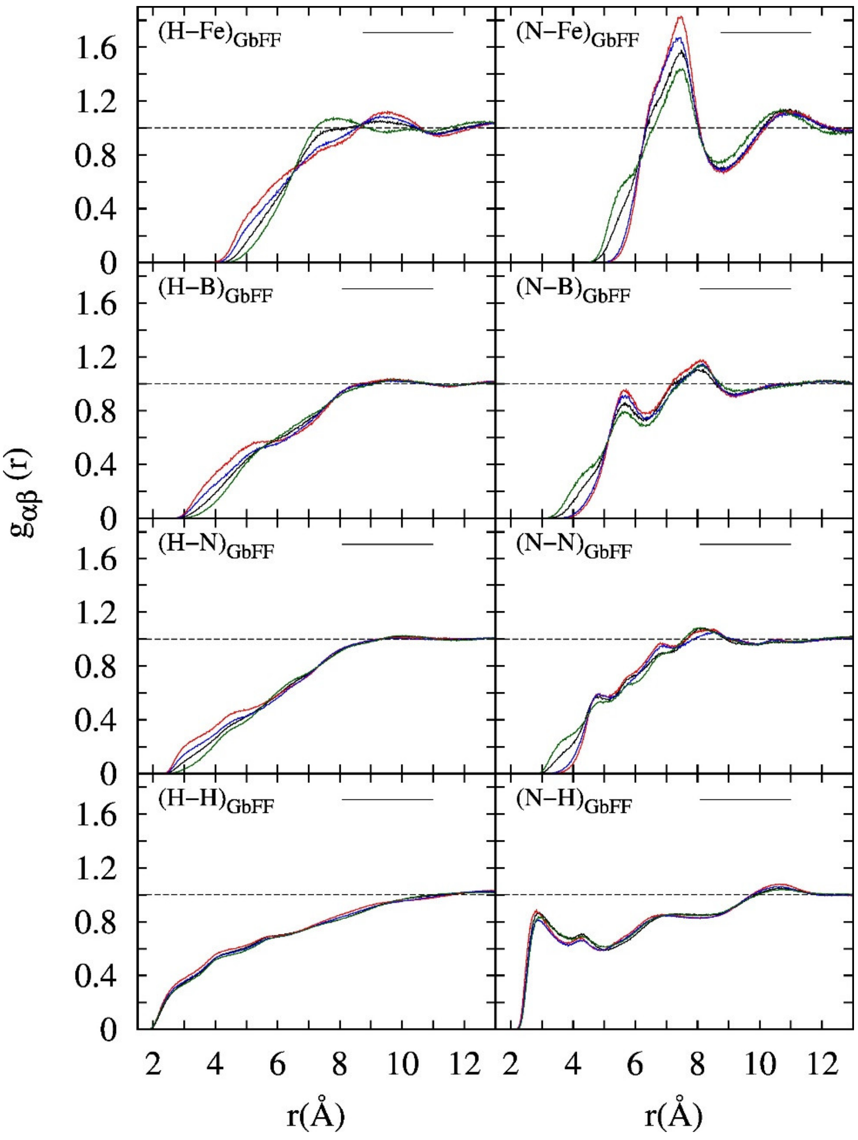

Figure S5. Atomic pair correlation functions $g_{\alpha\beta}$, computed with RESP (black), NPA (red), CM5 (blue) and ChelpG (green) atomic charges along the GbFF MD simulation runs, between the solute (β) atoms (Fe, B, N and H from $-CH_3$ group) of the complex as indicated in the (α-β) legend, and either the N (left part) or H (right part graphs) atoms of the acetonitrile solvent (α).

Table S1. Statistic data taken from the normal distribution of the (^{2}LMCT) band centers (λ_{max}). This distribution has been analyzed by considering all the TD-DFT individual spectra for each MD run snapshot studied in this work: average (μ) and standard deviations (σ) values in nm.

	**JOYCE**		**GbFF**	
**MM layer**	μ	σ	μ	σ
ACN	456.3	18.6	539.1	32.1
ACN+ion	456.5	16.9	527.6	37.7

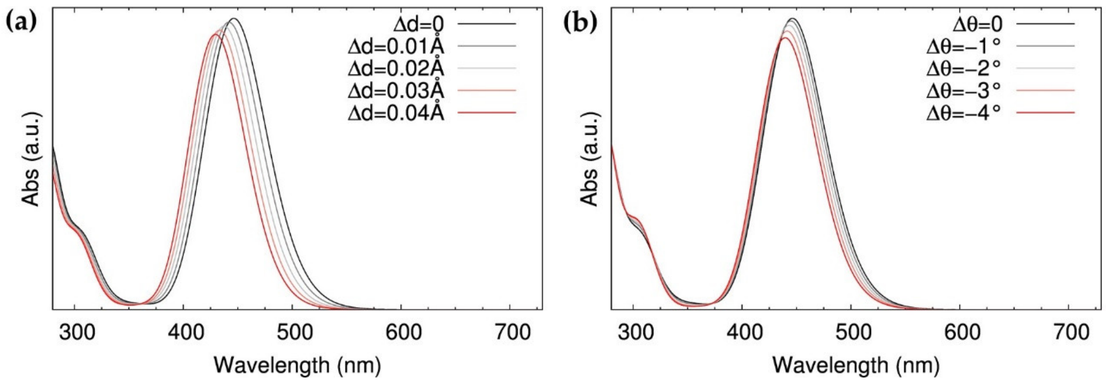

Figure S6. TD-DFT simulated spectra for the DFT GS complex geometries obtained upon the scan performed along the coordinates dictating the octahedral coordination: **(a)** the elongation of the Fe-C bonds (distance *d*); and **(b)** the bending of the C-Fe-C angles (θ angle) formed by the equivalent ligands. The geometry scan has been taken for each increment of 0.01Å in the Fe-C bond distances; and a decrease of 1° in the case of the C-Fe-C angles. Note that the change from GbFF based (red) to DFT and XRD (black color) equilibrium Fe-C distances and C-Fe-C angles, is translated in a red-shift in the spectra which already increases the absorption energies underestimation observed in the GbFF minimized structure spectrum (see Figure 7).

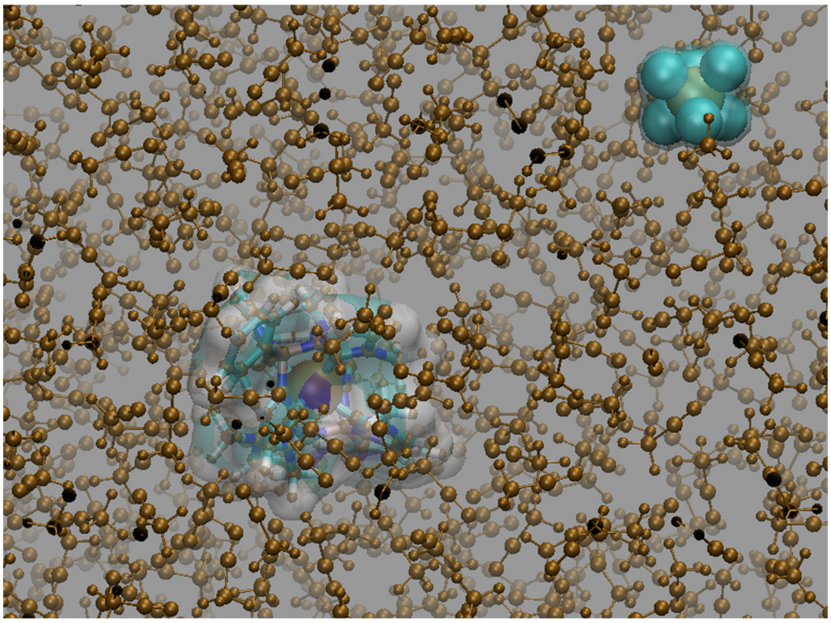

Figure S7. Snapshot of a large portion of the final equilibrated configuration of the solvated Fe complex (in the bottom, licorice representation) and its PF_6^- counter-ion (in top right corner with vdW spheres) in acetonitrile (balls&sticks) at 1 atm and 298 K.

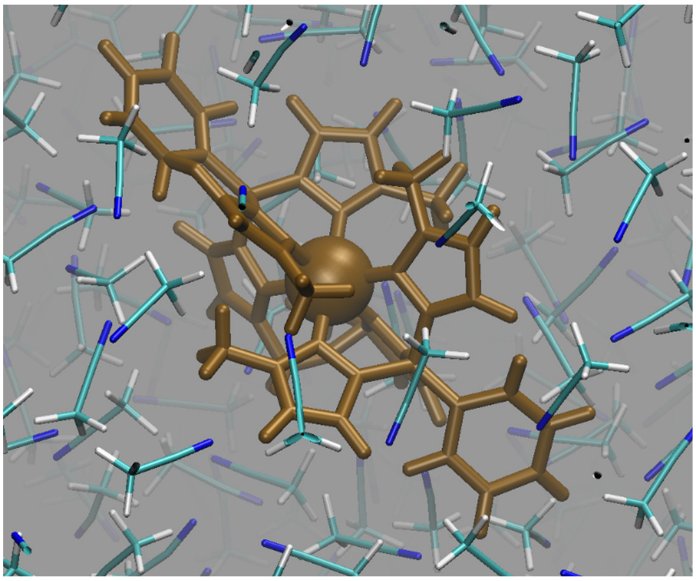

Figure S8. Zoom on the neighboring solvent molecules (licorice, Carbon, Nitrogen and Hydrogen are in cyan, white, and blue, respectively) around the Fe complex in its final equilibrated configuration.

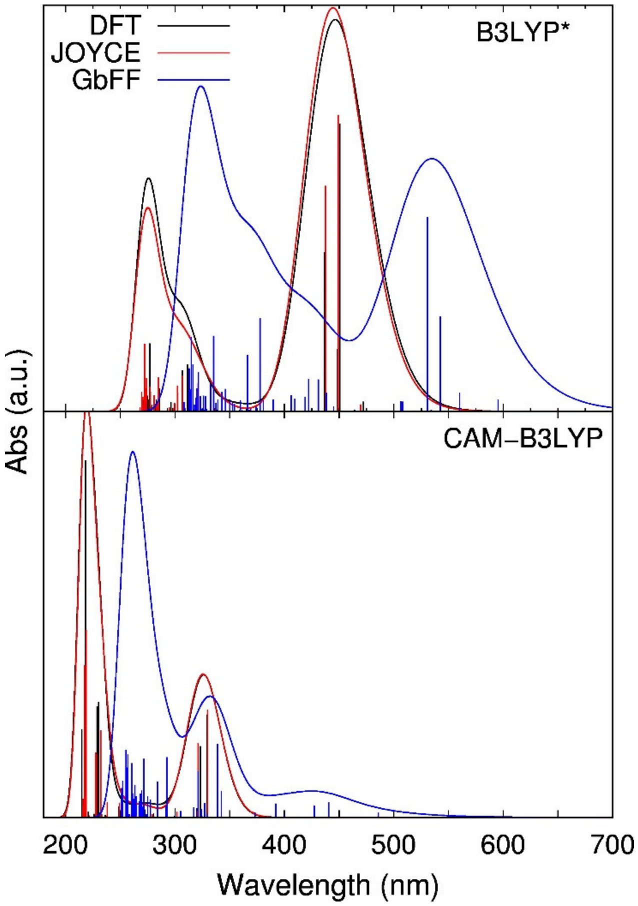

Figure S9. TD-DFT simulated spectra for the zero temperature complex structures obtained upon DFT (black), MM based JOYCE (red) and GbFF (blue) energy relaxations, as calculated by employing B3LYP* with 15% of Hartree-Fock exchange-correlation fraction (top panel) and CAM-B3LYP (bottom panel) functionals.
